# Crystal structure resolution of two different chlorhexidine salts

**DOI:** 10.1016/j.molstruc.2016.04.077

**Published:** 2016-10-05

**Authors:** Damiano Cattaneo, Laura J. McCormick, David B. Cordes, Alexandra M.Z. Slawin, Russell E. Morris

**Affiliations:** EaSTCHEM School of Chemistry, University of St Andrews, North Haugh, St Andrews, Fife, Scotland, KY16 9ST, UK

**Keywords:** Chlorhexidine, Crystal structure, Hydrogen bonds

## Abstract

Two salts of the chlorhexidine di-cation (H_2_CHx^2+^) – (H_2_CHx)(SO_4_)·3H_2_O and (H_2_CHx)(CO_3_)·4H_2_O – have been synthesised and characterised crystallographically.

## Highlights

• Crystals of two salts of the chlorhexidine dication have been prepared.
• The protonation sites on the chlorhexidine cation are different between the two salts.
• The cation and anion packing is very similar for both salts.
• Different hydrogen-bonding interactions occur between the CO_3_^2−^ and SO_4_^2−^ salts.

## 1 Introduction

Chlorhexidine (**I**, CHx) is a chemical disinfectant and antiseptic with a broad spectrum of action; it is active against Gram positive and Gram negative bacteria, as well as fungi [1]. It is a symmetrical bisbiguanidine, which is a class of chemically related compounds studied for their bactericidal properties. [1], [2] In the last 60 years chlorhexidine has been used as an antiseptic for mucous membranes, skin and wounds, or as a preservative in pharmaceutical formulations of ophthalmic products [2]. Due to its low solubility and ability to form micelles in solution [3], chlorhexidine does not crystallize easily, however, three salts of the chlorhexidine di-cation with anionic calixarenes were characterised crystallographically and reported in 2008 [4].

Herein we report the crystallographic characterisation of two salts of the chlorhexidine di-cation, namely (H_2_CHx)(SO_4_)·3H_2_O and (H_2_CHx)(CO_3_)·4H_2_O.

**Figure:**
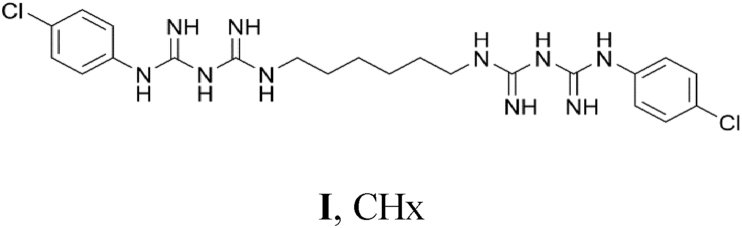

## 2 Results and discussion

### 2.1 Crystal structures of as-synthesised (H_2_CHx)(SO_4_)·3H_2_O and (H_2_CHx)(CO_3_)·4H_2_O

**Fig. 1:**
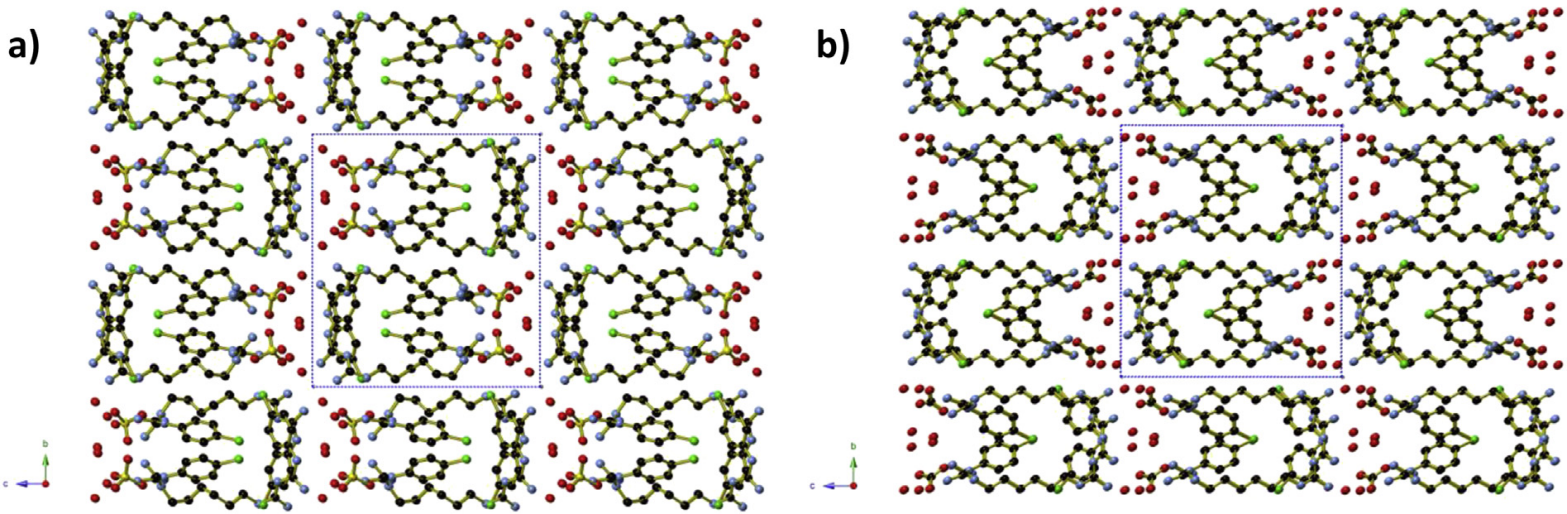
A view along the *a*-axis of **a)** (H_2_CHx)(SO_4_)·3H_2_O and **b)** (H_2_CHx)(CO_3_)·4H_2_O, showing the structural similarities between the two compounds.

**Fig. 2:**
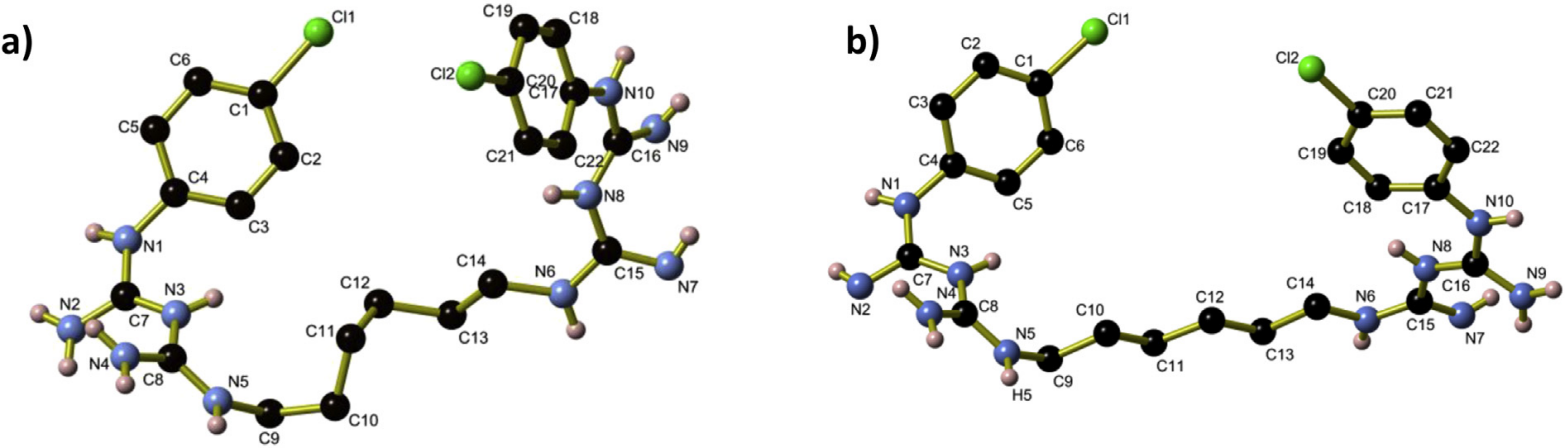
A view along the chlorhexidine cation found in **a)** (H_2_CHx)(SO_4_)·3H_2_O and **b)** (H_2_CHx)(CO_3_)·4H_2_O. Aromatic and aliphatic hydrogen atoms have been omitted for clarity.

**Fig. 3:**
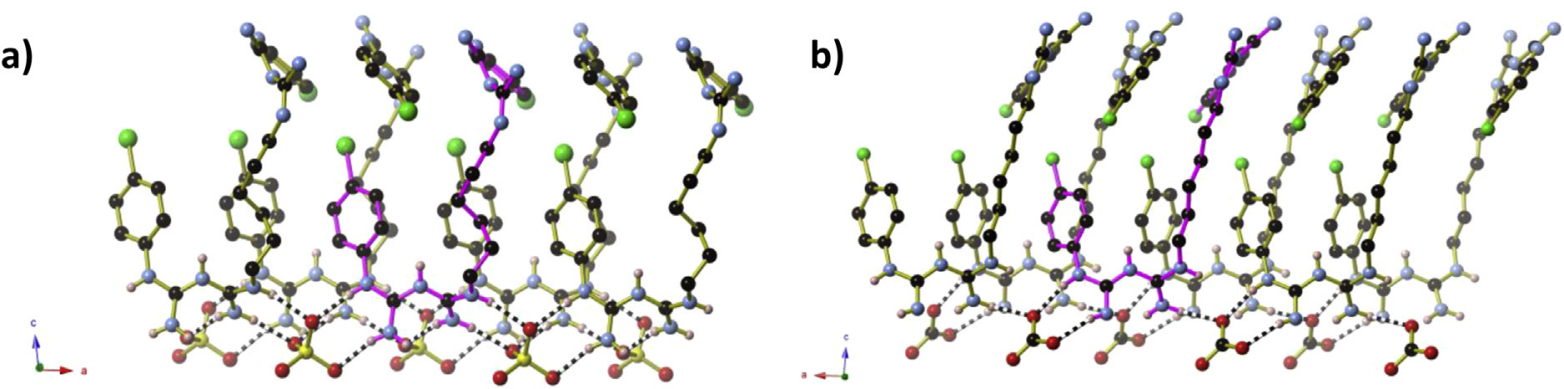
The hydrogen bonds between the H_2_CHx^2+^ coils and the anions in **a)** (H_2_CHx)(SO_4_)·3H_2_O and **b)** (H_2_CHx)(CO_3_)·4H_2_O. One H_2_CHx^2+^ cation has been highlighted in pink in each image. Hydrogen atoms not belonging to the biguanidine unit that forms hydrogen bonds have been omitted for clarity. (For interpretation of the references to colour in this figure legend, the reader is referred to the web version of this article.)

Crystals were prepared by reaction of neutral chlorhexidine and sodium sulfate or potassium carbonate in aqueous ethanol, as part of a series of attempts to generate chlorhexidine-containing coordination complexes. Full crystallographic details of both salts are presented in Table 1. Structurally, the two salts are very similar to one another (Fig. 1), with small variations in the conformation of the hexyl chains and intermolecular hydrogen-bonding due to the different arrangement of oxygen atoms between the tetrahedral SO_4_^2−^ and the trigonal planar CO_3_^2−^ anions, and different protonation sites along the chlorhexidine moiety. The differences in protonation site may be seen by a comparison of Fig. 2a and 2b: in (H_2_CHx)(SO_4_)·3H_2_O, one biguanidine moiety is doubly protonated, whilst the other remains unprotonated; in (H_2_CHx)(CO_3_)·4H_2_O, both biguanidine moieties are singly protonated in an asymmetrical manner. In both instances, the C—N bond lengths within the biguanidine units (1.307 Å to 1.379 Å) indicate that some delocalisation of the double and single bonds is occurring. The protonation of the biguanidine moieties of the chlorhexidine was unexpected, given the alkaline nature of the reaction solution. Chlorhexidine di-cations in both salts adopt a spiral conformation and are arranged into U-shaped ‘coils’ that extend parallel to the *a*-axis. In the labelling scheme in Fig. 2, the biguanidine moiety N1 to N5 lies at the open end of the U-shaped coils. Within the coils, adjacent H_2_CHx^2+^ cations alternate between left- and right-handed conformations, so that each coil is not helical overall (Fig. 3). The coils are held together by hydrogen bonds to the sulfate or carbonate anions and water molecules that occupy the spaces between coils.

**Fig. 4:**
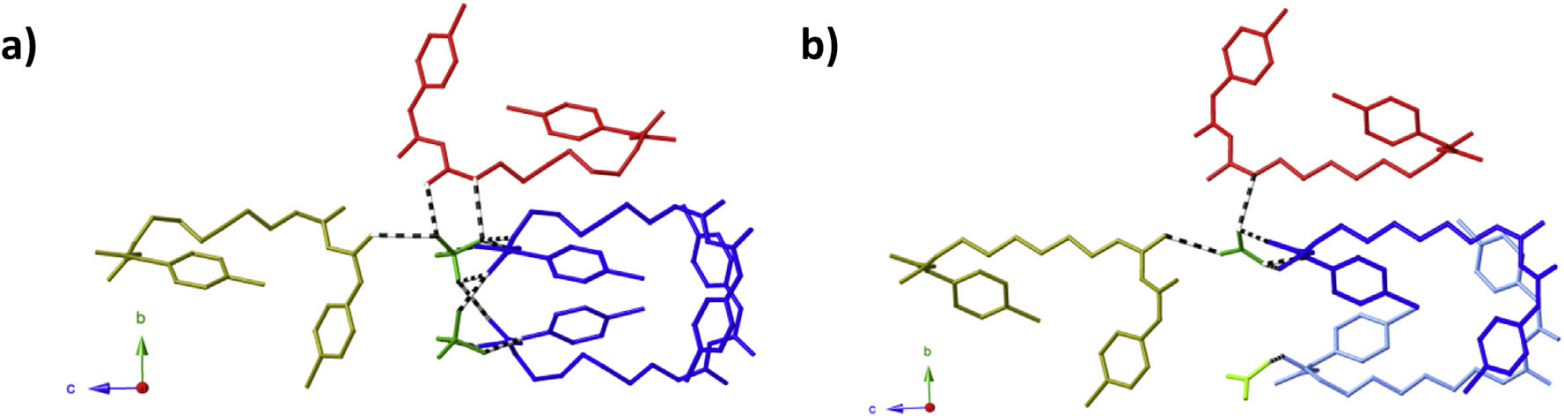
The hydrogen bonds between the oxyanions in **a)** (H_2_CHx)(SO_4_)·3H_2_O and **b)** (H_2_CHx)(CO_3_)·4H_2_O. Chlorhexidine cations shown in blue, red and gold belong to three different ‘coils’. Oxyanions are shown using green bonds. In **b)**, lighter bond colours have been used to show chlorhexidine and carbonate anions that belong to the second half of the central blue ‘coil’. (For interpretation of the references to colour in this figure legend, the reader is referred to the web version of this article.)

**Fig. 5:**
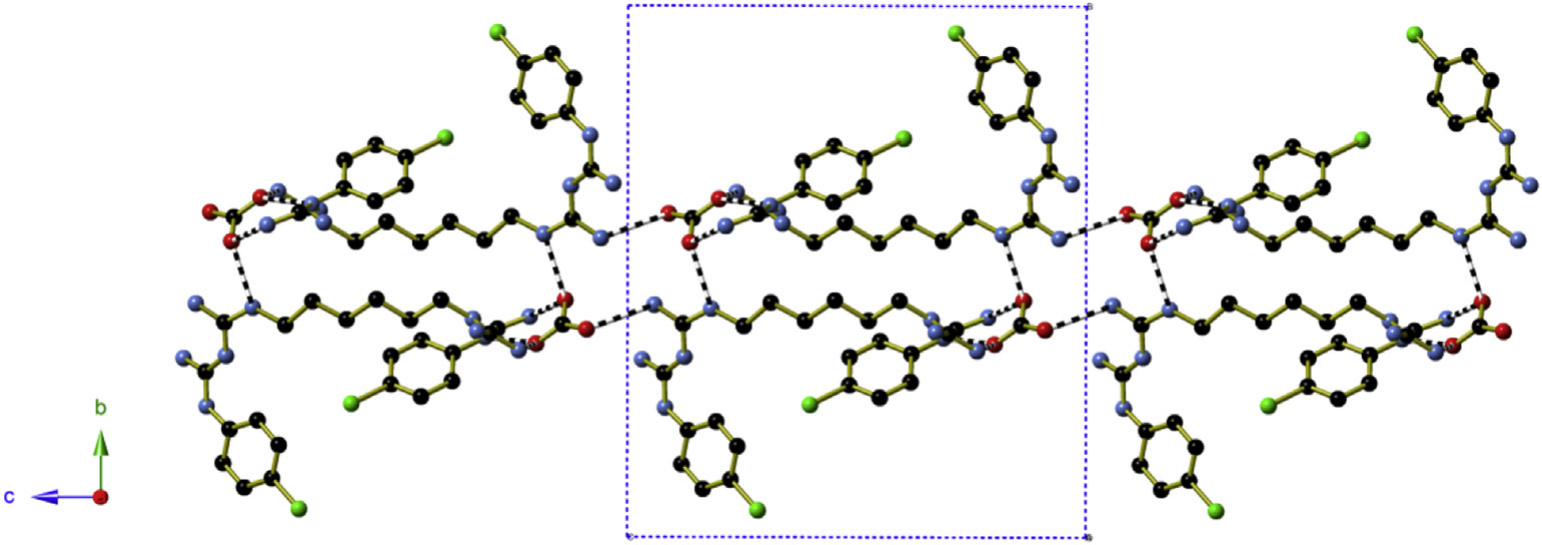
A view along the edge of the hydrogen-bonded sheet within (H_2_CHx)(CO_3_)·4H_2_O.

Each oxyanion participates in hydrogen-bonding interactions with chlorhexidine cations from three different coils. Carbonate anions are involved in hydrogen bonds with two chlorhexidine cations from one coil, in addition to one cation from each of two adjacent coils as shown in Fig. 4. Sulfate anions form hydrogen bonds with three cations from one coil, plus one cation from each of two adjacent coils. Due to the difference in shape of the SO_4_^2−^ and CO_3_^2−^ anions, the hydrogen-bonding interactions within the coils are slightly different. The SO_4_^2−^ anion forms hydrogen bonds to three adjacent chlorhexidine cations of both left- and right-handed conformation. When viewed along the *a*-axis, the sulfate anions are located towards the centre of the open end of the U-shaped coil and can interact with both halves of the U-shape. This generates a three-dimensional hydrogen-bonded framework of SO_4_^2−^ anions and H_2_CHx^2+^ cations. The CO_3_^2−^ anion, however, can participate in intra-chain hydrogen bonds with two non-adjacent chlorhexidine cations that both have the same conformation (either left- or right-handed). When viewed along the *a*-axis, the carbonate anions sit almost aligned with the two sides of the U-shape, and so are only able to interact with the chlorhexidine cations along one side of the U-shape. This generates a two-dimensional hydrogen-bonded framework of CO_3_^2−^ anions and H_2_CHx^2+^ cations that extends parallel to the *ac*-plane (Fig. 5). Water molecules of crystallisation occupy the remaining space between the coils and form hydrogen bonds with both the oxyanions and the chlorhexidine cations so that both compounds contain a complex three-dimensional hydrogen-bonded network. A list of the hydrogen bonds found in both compounds presented in Supporting Information.

## 3 Conclusions

The carbonate and sulfate salts of the chlorhexidine di-cation have been synthesised and characterised crystallographically. Investigations showed that the two salts are structurally similar to each other, although differences in the conformations of the hexyl chain and in the hydrogen-bonding interactions surrounding the cations were observed.

## 4 Experimental

All reagents for synthesis were purchased from Sigma-Aldrich, Fluka, and ABCR and were used without further purification.

### 4.1 Synthesis

#### 4.1.1 (H_2_CHx)(SO_4_)·3H_2_O

An aqueous solution (2.5 mL) of sodium sulfate (25 mg, 176 μmol) was added to a solution of chlorhexidine (15 mg, 30 μmol) in water/ethanol (5 mL, 1:1). A small number of colourless crystals formed after two weeks standing undisturbed at room temperature.

#### 4.1.2 (H_2_CHx)(CO_3_)·4H_2_O

An aqueous solution (2.5 mL) of potassium carbonate (25 mg, 181 μmol) was added to a solution of chlorhexidine (15 mg, 30 μmol) in water/ethanol (5 mL, 1:1). A small number of colourless crystals formed after two weeks standing undisturbed at room temperature.

### 4.2 Crystallography

Crystals were coated with a protective oil and mounted on a loop crystal mount. Crystallographic data were collected on a Rigaku Cu MM007 HF (dual port) high brilliance generator using a Dectris Pilatus P100 detector and Oxford DTC LT system. Absorption corrections were applied using multi-scan methods [5]. Structure solutions were obtained using SHELXS-97 and refined by full matrix on *F*^2^ using SHELXL-97 [6] and SHELXL-2014 [7] as part of the WinGX suite [8]. All full occupancy non-hydrogen atoms were refined with anisotropic thermal displacement parameters. Aromatic and aliphatic hydrogen atoms were included at their geometrically estimated positions. Hydrogen atoms belonging to full occupancy water molecules of crystallisation were assigned, fixed at a distance of 0.9 Å from the oxygen atom and 1.47 Å from the second hydrogen atom of the water molecule, and their thermal parameters linked to those of the oxygen atom to which they are bound. Similarly, the amine and imine hydrogen atoms were assigned, fixed at a distance of 0.88 Å from the nitrogen atom and (where appropriate) 1.52 Å from the second hydrogen atom of the protonated imine group, and their thermal parameters linked to the nitrogen atom to which they are bound. Crystallographic data are presented in Table 1.

## Figures and Tables

**Table 1:** Crystallographic details for (H_2_CHx)(SO_4_)·3H_2_O and (H_2_CHx)(CO_3_)·4H_2_O.

	(H_2_CHx)(SO_4_)·3H_2_O	(H_2_CHx)(CO_3_)·4H_2_O
Empirical formula	C_22_H_38_Cl_2_N_10_O_7_S	C_23_H_40_Cl_2_N_10_O_7_
Molecular weight	657.58	639.55
Temperature (K)	173(2)	173(2)
Wavelength (Å)	1.54187	1.54187
Crystal system	Monoclinic	Monoclinic
Space group	*P*2_1_/*a*	*P*2_1_/*a*
*a* (Å)	9.349(4)	8.497(2)
*b* (Å)	19.270(8)	21.0858(10)
*c* (Å)	17.365(7)	17.738(5)
β (°)	98.105(7)	91.930(5)
*V* (Å^3^)	3097(2)	3176.2(12)
*Z*	4	4
ρ (g cm^−1^)	1.410	1.337
μ (mm^−1^)	3.011	2.321
*F*(000)	1384	1352
*GooF*	1.182	1.124
Reflections/data/parameters	30743/5566/433	31375/5718/440
*R*_int_	0.0984	0.0875
Final *R* indices (*I* > 2σ(*I*))	*R*1 = 0.1061	*R*1 = 0.0642
w*R*2 = 0.2772	w*R*2 = 0.1411
Final *R* indices (all data)	*R*1 = 0.1288	*R*1 = 0.1026
w*R*2 = 0.3047	w*R*2 = 0.1705

## Appendix A Supplementary data

CCDC 1416048 and 1416049 contain the supplementary crystallographic data for this paper. These data can be obtained free of charge from The Cambridge Crystallographic Data Centre via www.ccdc.cam.ac.uk/data_request/cif.
