# Supplementary material for: Crystal structure resolution of two different chlorhexidine salts
Source: J Mol Struct. 2016 Oct 5;1121:70–3. doi: 10.1016/j.molstruc.2016.04.077 (PMC4920644; doi:10.1016/j.molstruc.2016.04.077)
Supplement: Supplementary file 1 [file mmc1.docx]

**Crystal structure resolution of two different chlorhexidine salts Supporting Information**

Damiano Cattaneo, Laura J. M^c^Cormick, David B. Cordes, Alexandra M. Z. Slawin and Russell E. Morris^[[1]](#footnote-1)^*

**S1.** Tables of hydrogen bonds

**S2.** Further details on the structural determinations

## S1 Hydrogen bond tables

**Table S1:** Hydrogen bonds for (H_2_CHx)(SO_4_)⋅3H_2_O [Å and °].

D-H···A d(D-H) d(H···A) d(D···A) <(DHA)

C(14)-H(14B)⋅⋅⋅Cl(2)^I^ 0.99 3.26 3.804(10) 116.3

N(6)-H(6N)⋅⋅⋅O(3) 0.877(10) 2.166(17) 3.028(5) 168(5)

O(1W)-H(1A)⋅⋅⋅O(2) 0.895(10) 2.006(16) 2.888(5) 168(5)

N(1)-H(1N)⋅⋅⋅O(3)^II^ 0.874(10) 2.03(2) 2.854(5) 157(5)

N(2)-H(2M)⋅⋅⋅O(2)^II^ 0.880(10) 2.067(18) 2.922(5) 164(4)

N(5)-H(5N)⋅⋅⋅O(3)^III^ 0.878(10) 2.08(3) 2.908(5) 156(5)

N(2)-H(2N)⋅⋅⋅O(2W)^III^ 0.880(10) 2.162(17) 3.018(6) 164(4)

N(9)-H(9N)⋅⋅⋅O(4)^IV^ 0.879(10) 2.119(19) 2.975(6) 164(5)

N(4)-H(4N)⋅⋅⋅O(1)^III^ 0.877(10) 2.056(12) 2.932(5) 176(4)

N(10)-H(10N)⋅⋅⋅O(3W)^IV^ 0.878(10) 2.177(12) 3.054(6) 177(5)

N(7)-H(7N)⋅⋅⋅O(1W)^IV^ 0.876(10) 2.13(2) 2.962(6) 157(5)

N(4)-H(4M)⋅⋅⋅O(1)^V^ 0.875(10) 2.116(15) 2.981(5) 169(4)

O(3W)-H(3A)⋅⋅⋅O(2)^VI^ 0.902(10) 2.05(3) 2.854(5) 148(4)

O(1W)-H(1B)⋅⋅⋅O(2W)^VII^ 0.896(10) 2.016(16) 2.901(6) 169(5)

O(2W)-H(2A)⋅⋅⋅O(4) 0.906(10) 2.41(5) 2.944(5) 118(5)

Symmetry transformations used to generate equivalent atoms:

**I:** x+1/2, -y+3/2, z **II:** -x+1, -y+2, -z+1 **III:** -x, -y+2, -z+1

**IV:** -x, -y+2, -z **V:** -x+1/2, y-1/2, -z+1 **VI:** x-1/2, -y+5/2, z

**VII:** x+1, y, z

**Table S2:** Hydrogen bonds for (H_2_CHx)(CO_3_)⋅4H_2_O [Å and °].

D-H···A d(D-H) d(H···A) d(D···A) <(DHA)

O(1W)-H(1A)⋅⋅⋅O(4W)^I^ 0.902(10) 1.97(2) 2.841(4) 161(5)

O(2W)-H(2B)⋅⋅⋅O(3W)^II^ 0.899(10) 1.882(11) 2.780(5) 179(4)

N(4)-H(4N)⋅⋅⋅O(3W)^II^ 0.879(10) 2.03(2) 2.865(4) 157(4)

N(7)-H(7N)⋅⋅⋅O(1C)^III^ 0.875(10) 1.957(12) 2.830(4) 175(5)

N(9)-H(9N)⋅⋅⋅O(1W)^III^ 0.883(10) 2.07(2) 2.874(4) 150(3)

N(1)-H(1N)⋅⋅⋅O(3C)^IV^ 0.873(19) 1.93(2) 2.801(4) 174(5)

N(2)-H(2N)⋅⋅⋅O(2C)^IV^ 0.874(10) 1.967(12) 2.839(4) 175(5)

N(6)-H(6N)⋅⋅⋅O(2C)^V^ 0.869(19) 1.99(2) 2.825(4) 162(5)

O(4W)-H(4B)⋅⋅⋅O(1W)^VI^ 0.902(10) 1.900(14) 2.790(4) 168(4)

N(9)-H(9M)⋅⋅⋅O(2W)^VII^ 0.883(10) 2.17(2) 2.961(4) 148(4)

N(10)-H(10N)⋅⋅⋅O(2W)^VII^ 0.890(19) 2.12(3) 2.958(4) 157(4)

N(4)-H(4M)⋅⋅⋅O(3C) 0.881(10) 1.952(14) 2.810(4) 164(4)

O(1W)-H(1B)⋅⋅⋅O(2C) 0.902(10) 1.810(15) 2.700(4) 169(5)

O(2W)-H(2A)⋅⋅⋅O(1C) 0.898(10) 1.806(12) 2.702(4) 174(5)

O(3W)-H(3A)⋅⋅⋅O(3C) 0.899(10) 1.855(15) 2.742(4) 168(5)

O(4W)-H(4A)⋅⋅⋅O(1C) 0.903(10) 1.915(14) 2.809(4) 170(5)

Symmetry transformations used to generate equivalent atoms:

**I:** x+1, y, z **II:** x-1/2, -y+3/2, z **III:** x, y, z-1 **IV:** x-1, y, z

**V:** -x+3, -y+1, -z+1 **VI:** -x+3, -y+1, -z+2 **VII:** x+1/2, -y+3/2, z-1

## S2 Further details on structure determinations

All C, N and Cl atoms belonging to the chlorhexidine cation, and all atoms belonging to the oxyanion and water molecules of crystallisation were assigned. Aliphatic and aromatic hydrogen atoms were included at their geometrically estimated positions, with thermal parameters fixed at 1.2 times that of the C atom to which they are bound. Hydrogen atoms belonging to full occupancy water molecules of crystallisation were assigned, fixed at a distance of 0.9 Å from the oxygen atom and 1.47 Å from the second hydrogen atom of the water molecule, and their thermal parameters fixed at 1.5 times that of the oxygen atoms to which they are bound.

*Locating imine and amine hydrogen atoms in (H_2_CHx)(SO_4_)·3H_2_O*

Six peaks of electron density (0.44 to 0.82 e.Å^-3^), located between 0.74 Å and 0.93 Å from the four imine nitrogen atoms (N2, N4, N7 and N9), were assigned as imine hydrogen atoms. Two such peaks of electron density were located near each of N2 and N4, whilst N7 and N9 were each close to only one such peak. These were fixed at a distance of 0.88 Å from the nitrogen atom and (where appropriate) 1.52 Å from the second hydrogen atom of the doubly protonated imine group, and their thermal parameters linked to the nitrogen atom to which they are bound.

Electron density peaks were located at similar distances to five of the six amine nitrogens. These were assigned as amine hydrogen atoms and fixed at a distance of 0.88 Å from the amine nitrogen atoms N1, N5, N6, N8 and N10. A similar peak near N3 could not be located, so the amine hydrogen atom was included at its geometrically estimated position. The SHELX AFIX command was then deleted, the N-H bond distance fixed at 0.88 Å and the coordinates of the hydrogen atom allowed to refine. The thermal parameters of all amine hydrogens were linked to that of the nitrogen atom to which they are bound.

*Locating imine and amine hydrogen atoms in (H_2_CHx)(CO_3_)·4H_2_O*

Two peaks of electron density (0.53 to 0.60 e.Å^-3^) were located between 0.87 Å and 0.95 Å from each of the four imine nitrogen atoms (N2, N4, N7 and N9). Two hydrogen atoms were included on each of the four imine nitrogen atoms at their geometrically estimated positions, and the hydrogen bonds surrounding these atoms were investigated to determine which two of the four imine groups are protonated. Only one of the protons bound to N7 was directed towards a hydrogen bond acceptor, indicating that N7 is not doubly protonated. The three remaining imine nitrogen atoms appear to participate in two hydrogen bonds each, however, one of these hydrogen bonds surrounding N2 has an N···O separation of approximately 3.03 Å, which we deem slightly too long to be a hydrogen bond given that the N⋅⋅⋅O separations of the hydrogen bonds surrounding N4 and N9 all lie in the 2.810(4) Å to 2.961(4) Å range. Thus N4 and N9 are assumed to be doubly protonated, and N2 and N7 only singly protonated. The calculated hydrogen atoms were then deleted and the peaks of electron density corresponding to the correct hydrogen atom locations were assigned. These were fixed at a distance of 0.88 Å from the nitrogen atom and (where appropriate) 1.52 Å from the second hydrogen atom of the doubly protonated imine group, and their thermal parameters linked to that of the nitrogen atom to which they are bound.

Peaks of electron density between 0.7 and 0.9 Å from the amine nitrogen atoms (N1, N3, N5, N6, N8 and N10) were assigned as hydrogen atoms and fixed at an N-H distance of 0.88 Å, and their thermal parameters linked to that of the nitrogen atom to which they are bound.

1. † EaSTCHEM School of Chemistry, University of St Andrews, North Haugh, St Andrews, Fife, Scotland, KY16 9ST, UK. E-mail: [rem1@st-andrews.ac.uk](mailto:rem1@st-andrews.ac.uk); Fax: +44 (0)1334 463808; Tel: +44 (0)1334 463800 [↑](#footnote-ref-1)
